# COVID-19 fatality and DALYs, and associated metabolic disorders and ambient air pollutants in pre-Omicron era of the pandemic: an international comparative study

**DOI:** 10.1265/ehpm.23-00350

**Published:** 2024-04-09

**Authors:** Nlandu-Roger Ngatu, Daniel-Kuezina Tonduangu, Emmanuel Munyeshyaka, Tomohiro Hirao, Georges-Matondo Balenda, Yusuke Yamadori, Takayuki Deguchi, Berthier Nsadi-Fwene, Jose-Nzunzu Lami, Steeve Akumwami, Kanae Kanda, Akitsu Murakami, Marie-Claire Yandju, Dieudonne-Tshipukane Nyembue, Antoine Tshimpi, Stanislas-Okitotsho Wembonyama

**Affiliations:** 1 Department of Public Health, Kagawa University Faculty of Medicine, Kagawa, Japan; 2 Department of Internal Medicine, Faculty of Medicine, University of Kinshasa, Democratic Republic of Congo (DRC); 3 Department of Microbiology, Kagawa University Faculty of Medicine, Kagawa, Japan; 4 Surgery Unit, Louis Pasteur Hospital, Pretoria, South Africa; 5 Department of Surgery, Faculty of Medicine, University of Kinshasa, DRC; 6 Faculty of Pharmaceutical Sciences, University of Kinshasa, DRC; 7 Department of Anesthesiology, Kagawa University Faculty of Medicine, Kagawa, Japan; 8 Oncology and Palliative Care Center, Kagawa University Faculty of Medicine, Kagawa, Japan; 9 Department of Biology, Faculty of Sciences, University of Kinshasa, DRC; 10 Department of Otorhinolaryngology, Faculty of Medicine, University of Kinshasa, DRC; 11 School of Public Health, University of Goma, D.R. Congo

**Keywords:** Air pollution, COVID-19, Disability-adjusted life years, Metabolic disorder

## Abstract

**Background:**

Air pollution and a number of metabolic disorders have been reported to increase the risk of severe COVID-19 outcomes. This study explored the association between severe COVID-19 outcomes, metabolic disorders and environmental air pollutants, at regional level, across 38 countries.

**Methods:**

We conducted an ecological study using COVID-19 data related to countries of the Organization for Economic Cooperation and Development (OECD), with an estimated population of 1.4 billion. They were divided into 3 regions: 1. Europe & Middle east; 2. Americas (north, central & south America); 3. East-Asia & West Pacific. The outcome variables were: COVID-19 case-fatality rate (CFR) and disability-adjusted life years (DALYs) at regional level. Freely accessible datasets related to regional DALYs, demographics and other environmental pollutants were obtained from OECD, WHO and the World in Data websites. Generalized linear model (GLM) was performed to determine the regional determinants of COVID-19 CFR and DALYs using the aggregate epidemiologic data (Dec. 2019–Dec. 2021).

**Results:**

Overall cumulative deaths were 65,000 per million, for mean CFR and DALYs of 1.31 (1.2)% and 17.35 (2.3) years, respectively. Globally, GLM analysis with adjustment for elderly population rate, showed that COVID-19 CFR was positively associated with atmospheric PM_2.5_ level (beta = 0.64(0.0), 95%CI: 0.06–1.35; p < 0.05), diabetes prevalence (beta = 0.26(0.1), 95%CI: 0.12–0.41; p < 0.001). For COVID-19 DALYs, positive associations were observed with atmospheric NOx level (beta = 0.06(0.0), 95%CI: 0.02–0.82; p < 0.05) and diabetes prevalence (beta = 0.32(0.2), 95%CI: 0.04–0.69; p < 0.05). At regional level, adjusted GLM analysis showed that COVID-19 CFR was associated with atmospheric PM_2.5_ level in the Americas and East-Asia & Western Pacific region; it was associated with diabetes prevalence for countries of Europe & Middle east and East-Asia & Western Pacific region. Furthermore, COVID-19 DALYs were positively associated with atmospheric PM_2.5_ and diabetes prevalence for countries of the Americas only.

**Conclusion:**

These findings confirm that diabetes and air pollution increase the risk of disability and fatality due to COVID-19, with disparities in terms of their impact. They suggest that efficient preventive and management programs for diabetes and air pollution countermeasures would have curtailed severe COVID-19 outcome rates.

## 1. Introduction

Metabolic disorders such as obesity, metabolic syndrome and diabetes are known as high-risk factors for severe COVID-19 outcomes. It has been reported that over 30% of people with COVID-19 who require hospitalization suffer from diabetes. Additionally, since the beginning COVID-19 pandemic, obesity has caused concerns given its high prevalence among infected patients [1, 2]. Furthermore, environmental or ambient air pollution has been reported to be the major environmental health risk factor worldwide. Air pollutants include particulate matters (PM), nitrite oxide (NOx), ozone (O3), sulfur oxide (SOx) and carbon monoxide (CO) [3]. They cause lung damage due increased oxidative stress, and endothelial dysfunction in the lung [4]. Thus, air pollutants are likely to aggravate the infection caused by SARS-CoV-2, the virus that is responsible of COVID-19.

A recent systematic review that included 116 studies showed that exposure to ambient O_3_, CO, PM_10_, PM_2.5_ and NO_2_ were associated with COVID-19, and the two-last pollutants were found to be the predictors of COVID-19 mortality [5].

Previous studies have shown that even short-term exposure to air pollution increases the susceptibility to SARS-CoV-2 infection [6, 7]. More recently, a genetic association study that employed the mendelian randomization to explore the relationship between cardiometabolic risk biomarkers and severe SARS-CoV-2 infection has incriminated obesity and diabetes as contributors in the occurrence of severe COVID-19 outcomes such as intensive care unit admissions and deaths [8]. However, studies that explored the association between COVID-19 severe outcomes (disease case fatality or disability-adjusted life years, DALYs) at regional level and/or air pollution are scarcely found in the medical literature. In the present study, we explored the associations between severe COVID-19 case fatality (CFR) and DALYs on one hand, and lifestyle-related metabolic disorders (obesity, diabetes) and air pollutants (NOx, PM_2.5_) on the other hand, in the pre-Omicron phase of the pandemic (2019–2021) across member states of the Organization for Economic Cooperation and Development (OECD).

## 2. Materials and methods

### 2.1. Study design and population

This was an ecological study COVID-19 using data of 38 countries belonging to the Organization for Economic Cooperation and Development (OECD). OECD countries were divided in three regions: region 1 comprising Europe & Middle east (Austria, Belgium, Czech Republic, Denmark, Estonia, Finland, France, Germany, Greece, Hungary, Iceland, Ireland, Israel, Italy, Latvia, Lithuania, Luxembourg, Netherlands, Norway, Poland, Portugal, Slovak Republic, Slovenia, Spain, Sweden, Switzerland, Turkey, England); region 2: the Americas comprising the north, central & south America (USA, Canada, Mexico, Costa Rica, Chile, Colombia); region 3 composed of East-Asia & West Pacific (Japan, South Korea, Australia, New Zealand). The estimated population of the 38 countries was 1.4 billion.

### 2.2. Outcome, explanatory variables and definitions

The outcome variables were: (1) COVID-19 case fatality rate (COVID-19 CFR) and (2) disability-adjusted life years (COVID-19 DALYs), which represent the sum of years of life lost due to premature death from a disease, and the years of healthy life lost due to disability caused by the disease. COVID-19 CFR and DALYs values used in the analysis are of 31 December 2021. CFR was calculated using cumulative COVID-19 cases and deaths statistics as reported previously [9]. Fully vaccinated individuals were those who received at least two doses of anti-COVID-19 vaccine.

CFR: in epidemiology, it is the proportion of people who die from a specified disease among all individuals diagnosed with the disease over a certain period of time.
DALYS: the sum of years of life lost due to premature death from a disease, and the years of healthy life lost due to disability caused by the disease.

The covariables were country-level demographic and health indicators (population, elderly population rate, full vaccination coverage per 100,000, overweight/obese population rate), a socioeconomic indicator (gross domestic product (GDP) *per capita*), metabolic disorders (overweight/obese population rate, diabetes prevalence) and environmental factors (air pollutants: nitrite oxides (NOx), particulate matter with a diameter of less than 2.5 micrometers (PM_2.5_)).

### 2.3. Ethical considerations, data sources and analysis

Data used in this study are anonymous, and informed consent was not necessary. Country-level socioeconomic, demographic characteristics and environmental or ambient air pollutants, including data on country level overweight/obesity rate, diabetes prevalence, regional level DALYs, were obtained from OECD statistics and Our World in Data databases [10, 11]. For data analysis, first of all Pearson correlation analysis was employed to investigate the statistical relationship between the outcome variables and the covariates. Thereafter, normality test (Shapiro-Wilk) was carried out, which showed that the continuous outcome variables were not normally distributed; thus, generalized linear model (GLM) was performed to determine the predictors of COVID-19 CFR and DALYs, with the use of aggregate epidemiologic data. Stata statistical software version 16 (StataCorp., TX, USA) was used for the analyses, and the significance level was set at p < 0.05.

## 3. Results

### 3.1. Demographics and health indicators in pre-Omicron phase of the pandemic

The region with highest rate of full anti-COVID-19 vaccination coverage (as of December 2021) was achieved in Europe & Middle-east region, 166.0 (SD: 26) per 10^5^ population. Highest ratio of overweight/bese population and diabetes prevalence rate were found in the Americas, 69.8 (SD: 7.4)% per 10^5^ and 10.5 (SD: 3.4)%, respectively. Regarding COVID-19 cases and deaths, the Americas was the most affected region with 163,458.7 (SD: 216,709.0) cases per 10^6^ and 2,912 (SD: 2,253.3) deaths per 10^6^ (Table 1).

**Table 1:** Regional distribution of demographics and COVID-19 health indicators (as of December 2021)

**Variables**	**Region**	**Mean (SD)**
Population (×10^3^)	Region1	20,951.5 (26,578)
	Region2	94,757.2 (122,479)
	Region3	52,017.8 (53,392)

Elderly population rate (%)	Region1	18.2 (3.2)
	Region2	12.0 (4.5)
	Region3	18.3 (6.5)

Full vaccination coverage/10^5^	Region1	166.7 (26)
	Region2	160.0 (39.9)
	Region3	162.0 (2.3)

Overweight & obese/10^3^	Region1	61.7 (4.3)
	Region2	69.8 (7.4)
	Region3	47.8 (20.2)

Diabetes prevalence rate/10^4^	Region1	6.2 (2.3)
	Region2	10.5 (3.4)
	Region3	6.5 (0.3)

COVID-19 cumulative cases per 10^6^	Region1	145,840.0 (48,349.5)
	Region2	163,458.7 (216,709)
	Region3	11,344.5 (5,977.8)

COVID-19 cumulative deaths/10^6^	Region1	1724.0 (922.2)
	Region2	2912.0 (2253.3)
	Region3	88.3 (57.5)

DALYS (in years)	Region1	16.5 (1.6)
	Region2	18.4 (2.2)
	Region3	22.00 (0)

Case fatality rate (CFR; %)	Region1	1.1 (0.6)
	Region2	2.4 (2.6)
	Region3	0.7 (0.3)

**-Notes:** DALYs, disability-adjusted life years; CFR, case-fatality rate; COVID-19, coronavirus disease 2019; region1, the Americas (north, central, south America); region 2, European and middle-east countries that are member states of OECD; region 3, Asia-Pacific region comprised of Asian and oceanian countries that are member states of OECD.

Furthermore, mean COVID-19 DALYs were highest in countries of the Asia & West Pacific (region 3), 22.0 years (SD: 0), followed by Europe & Middle east (region 1), 18.4 years (SD: 2.2) and the Americas (region 1), 16.5 years (SD: 1.6). Regarding COVID-19 fatality, highest rates were observed in Europe & Middle-east, 2.4% (SD: 2.6), followed by Europe & Middle east, 1.1% (SD: 0.6) (Table 1).

### 3.2. Relationship between metabolic disorders (overweight/obesity, diabetes) and COVID-2019 severe outcomes (CFR, DALYs) in the pre-Omicron phase of the pandemic at regional level

Table 2 shows health system, health indicators, metabolic conditions and air pollutants that were correlated with COVID-19 CFR and COVID-19 DALYs, as of December 2023. Diabetes prevalence was correlated with COVID-19 CFR (rho = 0.61; p = 0.000) and COVID-2019 DALYs (rho = 0.42; p = 0.009). Annual atmospheric PM_2.5_ level was positively correlated with COVID-19 CFR (rho = 0.47; p = 0.024), whereas annual NOx level was correlated with COVID-19 DALYs (rho = 0.43; p = 0.0132) (not shown).

**Table 2:** COVID-19 CFR determinants among OECD countries in pre-Omicron era by GLM analysis

**Determinants of COVID-19 CFR**	**Model 1**		**Model 2**	
	
	**Beta (SE)**	**95% CI**	**Beta (SE)**	**95% CI**
Elderly population rate	0.12 (0.5)	0.02–0.41	-	-
Full vaccination coverage rate	−0.21 (0.1)	−0.35–−0.06*	−0.19 (0.1)	−0.34–−0.05
Overweight/obese population rate	0.04 (0.0)	0.17–0.24	0.03 (0.1)	0.07–0.19
Atmospheric NOx (annual average)	0.03 (0.0)	0.01–0.12	0.1 (0.0)	−0.01–0.21
Atmospheric PM2.5 (annual average)	0.77 (0.3)	0.14–1.07*	0.64 (0.0)	0.06–1.35*
Diabetes prevalence	0.24 (0.1)	0.04–0.38^##^	0.26 (0.1)	0.12–0.41^##^

**-Notes**: *, p-value less than 0.05; #, p-value less than 0.01; ##, p-value less than 0.001; COVID-19, coronavirus disease 2019; CFR, case fatality rate; GLM, generalized linear model; OECD, Organization for Economic Cooperation and Development; CI, confidence interval; SE, standard error; NOx, nitrite oxides; PM2.5, particulate matter of less than 2.5 micrometers.

Model 1: analysis without adjustment; Model 2: analysis with adjustment for elderly population rate.

Results from the GLM analysis are shown in Table 3, Table 4 and Table 5. In model 1, which consisted in GLM analysis without adjustment, COVID-19 CFR was positively associated with annual atmospheric PM_2.5_ level (beta = 0.77 (SE: 0.3), 95%CI: 0.14–1.07; p < 0.05) and diabetes prevalence (beta = 0.24 (SE: 0.1), 95%CI: 0.04–0.38; p < 0.001); whereas it was negatively associated with full anti-COVID-19 vaccination coverage rate (beta = −0.21 (SE: 0.1), 95%CI: −0.35–−0.06; p < 0.05) (Table 2). In model 2 (GLM analysis with adjustment for elderly population rate), COVID-19 CFR was positively associated with atmospheric PM_2.5_ level (beta = 0.64 (0.0), 95%CI: 0.06–1.35; p < 0.05), and diabetes prevalence (beta = 0.26 (SE: 0.1), 95%CI: 0.12–0.41; p < 0.001) (Table 2).

**Table 3:** COVID-19 DALYs determinants among OECD countries in pre-Omicron era by GLM analysis

**Determinants of COVID-19 DALYs**	**Model 1**		**Model 2**	
	
	**Beta (SE)**	**95% CI**	**Beta (SE)**	**95% CI**
Elderly population rate	−0.55 (0.2)	−1.12–0.25	-	-
Full vaccination coverage rate	−0.97 (0.1)	−1.10–1.14	−0.57 (0.2)	−0.89–1.75
Overweight/obese population rate	0.16 (0.0)	0.07–0.40	0.13 (0.1)	0.05–0.31
Atmospheric NOx (annual average)	0.07 (0.0)	0.02–0.08^#^	0.06 (0.0)	0.02–0.82*
Atmospheric PM2.5 (annual average)	0.51 (0.4)	−0.35–1.37	0.19 (0.4)	−0.64–1.03
Diabetes prevalence	0.35 (0.1)	0.09–0.61^#^	0.32 (0.2)	0.04–0.69*

**-Notes**: *, p-value less than 0.05; #, p-value less than 0.01; ##, p-value less than 0.001; COVID-19, coronavirus disease 2019; DALYs, disability-adjusted life years; GLM, generalized linear model; OECD, Organization for Economic Cooperation and Development; CI, confidence interval; SE, standard error; NOx, nitrite oxides; PM2.5, particulate matter of less than 2.5 micrometers.

Model 1: analysis without adjustment; Model 2: analysis with adjustment for elderly population rate.

**Table 4:** Regional determinants of COVID-19 CFR by adjusted GLM analysis

**Determinants of COVID-19 CFR**	**Region 1**		**Region 2**		**Region 3**	
	
	**Beta (SE)**	**95% CI**	**Beta (SE)**	**95% CI**	**Beta (SE)**	**95% CI**
Full vaccination coverage rate	−0.1 (0.0)	−0.02–0.04	−0.2 (0.0)	−0.08–0.03	-	-
Overweight/obese population rate	0.2 (0.3)	0.10–0.21	0.5 (0.9)	0.13–0.43	0.1 (0.0)	0.04–0.31
Atmospheric NOx level	0.2 (0.0)	0.06–0.21	-	-	-	-
Atmospheric PM_2.5_ level	0.8 (0.0)	0.45–1.74^#^	0.3 (0.2)	0.07–0.10	0.4 (0.1)	0.17–0.68^#^
Diabetes prevalence rate	0.8 (0.6)	−0.45–1.61	1.4 (0.5)	0.27–1.57*	0.8 (0.0)	0.23–1.47^#^

**-Notes**: *, p-value less than 0.05; #, p-value less than 0.01; ##, p-value less than 0.001; COVID-19, coronavirus disease 2019; CFR, case fatality rate; GLM, generalized linear model; OECD, Organization for Economic Cooperation and Development; CI, confidence interval; SE, standard error; NOx, nitrite oxides; PM2.5, particulate matter of less than 2.5 micrometers.

**Table 5:** Regional determinants of COVID-19 DALYs by adjusted GLM analysis

**Determinants of COVID-19 DALYs**	**Region 1**		**Region 2**		**Region 3**	
Full vaccination coverage	0.3 (0.1)	−0.01–0.21	−0.3 (0.0)	−0.08–0.02	-	-
Overweight/obese population	0.1 (0.1)	0.20–0.43	0.8 (0.2)	0.17–0.42	-	-
Atmospheric NOx	0.1 (0.0)	−0.04–0.24	-	-	-	-
Atmospheric PM2.5	0.6 (0.4)	0.13–0.82*	0.7 (0.1)	0.47–0.83	-	-
Diabetes prevalence	0.5 (0.1)	0.15–0.94^#^	0.3 (0.1)	−0.07–0.47	-	-

**-Notes**: *, p-value less than 0.05; #, p-value less than 0.01; ##, p-value less than 0.001; COVID-19, coronavirus disease 2019; DALYs, disability-adjusted life years; case fatality rate; GLM, generalized linear model; OECD, Organization for Economic Cooperation and Development; CI, confidence interval; SE, standard error; NOx, nitrite oxides; PM2.5, particulate matter of less than 2.5 micrometers.

Regarding COVID-19 DALYs, in model 1, positive associations were observed with atmospheric NOx level (beta = 0.07(0.0), 95%CI: 0.02–0.08; p < 0.01) and diabetes prevalence (beta = 0.35(0.1), 95%CI: 0.09–0.61; p < 0.01). In model 2, the result remained the same for both atmospheric NOx level (beta = 0.06(0.0), 95%CI: 0.02–0.82; p < 0.05) and diabetes prevalence (beta = 0.32(0.2), 95%CI: 0.04–0.69; p < 0.05) (Table 3).

At regional level, GLM analysis adjusted for elderly population rate showed that COVID-19 CFR was associated with annual atmospheric PM_2.5_ level in the Americas (beta = 0.8(0.0), 95%CI: 0.45–1.74; p < 0.01) and East-Asia & West Pacific region (beta = 0.4(0.1), 95%CI: 0.17–0.68; p < 0.01); it was also associated with diabetes prevalence in Europe & Middle east region (beta = 0.7(0.5), 95%CI: 0.27–1.57; p < 0.05) and in East-Asia & Western Pacific region (beta = 0.8(0.0), 95%CI: 0.23–1.47; p < 0.01) (Table 4). Furthermore, COVID-19 DALYs was found to be positively associated with atmospheric PM2.5 (beta = 0.6(0.4), 95%CI: 0.13–0.82; p < 0.05) and diabetes prevalence (beta = 0.3(0.1), 95%CI: 0.15–0.94; p < 0.01) in the Americas only (Table 5).

## 4. Discussion

This study analyzed the relationship between COVID-19 CFR and DALYs on one hand, and metabolic disorders (overweight & obesity, diabetes) and air pollutants on the other hand, at regional level, across 38 OECD countries. When considering OECD countries globally, it was found that, globally, diabetes prevalence was positively associated with both COVID-19 CFR and COVID-19 DALYs in the pre-Omicron era (first and the second year) of the pandemic. Specifically, the latter association was more evident in the Americas. These findings are in line with observations from previous studies. An Iranian study [12] conducted among COVID-19 patients showed that those with severe outcomes had diabetes. A review of the literature by Lim and colleagues [13] also showed that diabetes was one of the risk factors for COVID-19 mortality. It is evident that hyperglycemia status can interact with other risk factors and modulate both the immune and inflammatory responses during SARS-CoV2 infection, which can lead to lethal outcomes [12].

Additionally, another review and meta-analysis conducted in the first year of the pandemic showed that obesity increased the risk of hospitalization and mortality among COVID-19 patients [14]. Furthermore, an international genetic study that included over 200,000 subjects found that diabetes was one of the factors that increased the risk of COVID-19-related death [8].

When considering all OECD countries, our study also showed that the annual averages of atmospheric PM_2.5_ was positively associated with both COVID-19 CFR, whereas NOx was associated only with COVID-19 DALYs. Air pollutants were found to be associated with severe COVID-19 outcomes mostly in the Americas and East-Asia & Western Pacific region. Recently, the EpiCovAir Study consducted by Stafoggia and colleagues [15] among 4 million Italian COVID-19 patients provided evidence on the association between long-term exposure to ambient air pollution and COVID-19 mortality. This study found that an estimated 8% of COVID-19 deaths attributable to air pollutant levels higher than exposure limit according to 2021 WHO air quality guidelines.

Our study also showed inverse associations between COVID-19 CFR and full vaccination coverage in the GLM analysis without adjustment for elderly population rate; but this association was not observed when the influence of age was taken into account. Disparities exist in terms of the effects of COVID-19 vaccination, as reported previously. A Canadian study conducted in 2021 found that antiCOVID-19 vaccine did reduce the disease mortality in Ontario; however, the authors suggested that age was the only significant factor associated with COVID-19 mortality [16]. A Japanese study by Ko et al. [17] showed that, among full vaccinated patients, vaccine effectiveness to prevent death varied according to the age of COVID-19 patients; it was 77.7% effective in patients aged over 90 years and higher (83.5–88.6%) in those aged 60–80 years. Thus, the protective effect of antiCOVID-19 immunization may vary, and more studies are needed to clarify the differences in the effectiveness of available vaccines.

The present study highlights factors associated with COVID-19 fatality and DALYs among OECD member states in the pre-Omicron era of the pandemic. The findings might be true only for groups of countries belonging to this organization; however, they may not be applicable to each individual OECD country or non OECD member states. Nonetheless, the findings may provide useful insights on potential predictors of severe outcomes of SARS-CoV-2 infection.

## 5. Conclusion

Findings from this study corroborate those reported previously in other parts of the world. Diabetes and air pollution were found to increase the likeliness of severe COVID-19 outcomes occurrence, especially the disease mortality and disability, among OECD countries. The implementation of efficient preventive and management programs for diabetes, combined with air pollution countermeasures, would have reduced COVID-19-related fatality and disability in severely affected regions.

## Declarations

### Availability of data and material

Data analyzed in this study can be obtained upon request to the corresponding author (NRN).
